# Influence of the pneumococcal conjugate vaccines on the temporal variation of pneumococcal carriage and the nasal microbiota in healthy infants: a longitudinal analysis of a case–control study

**DOI:** 10.1186/s40168-017-0302-6

**Published:** 2017-07-24

**Authors:** Moana Mika, Josua Maurer, Insa Korten, Aurélie Allemann, Suzanne Aebi, Silvio D. Brugger, Weihong Qi, Urs Frey, Philipp Latzin, Markus Hilty

**Affiliations:** 1 Institute for Infectious Diseases, University of Bern, Friedbühlstrasse 51, 3010 Bern, Switzerland; 2 Graduate School for Cellular and Biomedical Sciences, University of Bern, Bern, Switzerland; 3 Division of Respiratory Medicine, Department of Pediatrics, Inselspital, University of Bern, Bern, Switzerland; 4 Department of Microbiology, The Forsyth Institute, Cambridge, MA USA; 5 Department of Oral Medicine, Infection and Immunity, Harvard School of Dental Medicine, Boston, MA USA; 6 Functional Genomics Center, Swiss Federal Institute of Technology Zurich/University of Zurich, Zurich, Switzerland; 7 University Children’s Hospital (UKBB), Basel, Switzerland; 8 Department of Infectious Diseases, University Hospital, Bern, Switzerland

**Keywords:** Pneumococcal conjugate vaccine, Pneumococcal carriage, Nasal microbiota, Oligotyping, Healthy infants, Prospective cohort study

## Abstract

**Background:**

Bacterial colonization of the upper airways is a prerequisite for subsequent invasive disease. With the introduction of the 7- and 13-valent pneumococcal conjugate vaccines (PCV7 and PCV13), changes in pneumococcal upper airway colonization have been described. It is, however, less evident whether the vaccines lead to compositional changes of the upper airway microbiota. Here, we performed a case–control study using samples from a longitudinal infant cohort from Switzerland. We compared pneumococcal carriage and the nasal microbiota within the first year of life of healthy infants vaccinated with either PCV7 (*n* = 20, born in 2010) or PCV13 (*n* = 21, born between 2011 and 2013). Nasal swabs were collected every second week (*n* = 763 in total). Pneumococcal carriage was analyzed by quantitative PCR of the pneumococcal-specific *lytA* gene. Analysis of the bacterial core microbiota was performed based on *16S rRNA* sequencing and subsequent oligotyping. We exclusively performed oligotyping of the core microbiota members, which were defined as the five most abundant bacterial families (*Moraxellaceae*, *Streptococcaceae*, *Staphylococcaceae*, *Corynebacteriaceae*, and *Pasteurellaceae*). Linear mixed effect (LME) and negative binomial regression models were used for statistical analyses.

**Results:**

We found a higher number of samples positive for pneumococcal carriage in PCV7- compared to PCV13-vaccinated infants (LME model; *P* = 0.01). In contrast, infants vaccinated in the PCV13 era had an increased alpha diversity as measured by the richness and the Shannon Diversity Index (LME model; *P* = 0.003 and *P* = 0.01, respectively). Accordingly, the PCV13 era was associated with clusters of a higher diversity than PCV7-associated clusters. Furthermore, infants vaccinated with PCV13 had a higher binary-based within-subject microbiota similarity, as well as a decreased Jensen–Shannon distance over time as compared to PCV7-vaccinated infants, indicating a higher microbiota stability in the PCV13 era (LME model and *t* test; *P* = 0.06 and *P* = 0.03, respectively).

**Conclusions:**

We hypothesize that the higher diversity and stability of the upper airway microbiota in the PCV13 era is the result of the lower pneumococcal carriage rate. This seems to indicate that the nasal bacterial microbiota of infants has changed in recent years as compared to the beginning of this study.

**Electronic supplementary material:**

The online version of this article (doi:10.1186/s40168-017-0302-6) contains supplementary material, which is available to authorized users.

## Background

*Streptococcus pneumoniae* is a frequent asymptomatic colonizer of the infant’s nasopharynx [1]. However, pneumococcal carriage is a prerequisite for subsequent invasive disease, such as bacteremia, pneumonia, and meningitis [2, 3]. Due to this duality of commensalism and pathogenicity, *S. pneumoniae* is alternatively defined as a pathobiont [4].

The introduction of the pneumococcal conjugate vaccines (PCVs) has lowered the burden of invasive pneumococcal disease (IPD), non-bacteremic pneumococcal pneumonia, and pneumococcal otitis media in different countries worldwide [5]. Interestingly, PCVs have prevented a disproportionally high amount of otitis media disease episodes as compared with non-bacteremic pneumonia or IPD cases [6]. PCVs have mainly been used for infants; however, pneumococcal infections declined also in adults and elderly people, mostly through the effect of herd immunity [2, 7, 8]. It is therefore challenging to disentangle the effects of herd immunity from the direct effects of the vaccines. PCVs act by targeting the capsular polysaccharides of the most frequently isolated serotypes of *S. pneumoniae* in IPD [9]. The 7-valent PCV (PCV7), which covers seven different pneumococcal serotypes (serotypes 4, 6B, 9V, 14, 18C, 19F, and 23F), was recently replaced by the 13-valent PCV (PCV13) covering 13 different pneumococcal serotypes (PCV7 serotypes plus serotypes 1, 3, 5, 6A, 7F, and 19A). In Switzerland, PCV7 was introduced in late 2006 and subsequently replaced by PCV13 in 2011 [10]. Regarding pneumococcal carriage, it is not yet clear whether the PCVs led to a reduced carriage rate. Results from various countries are discordant; while some studies found a reduction in the overall pneumococcal carriage, others did not observe this effect [11, 12].

Among lowering the frequency of disease, cross-sectional studies demonstrated that PCV7 induced a change in the prevalence of species in the upper airway microbiota in infants and young children [13–15]. The relative abundance of the potential pathogens *Staphylococcus aureus* and *Haemophilus influenzae*, for example, was found increased in PCV7-vaccinated infants as compared to controls [15]. The upper airway microbiota of infants is although highly personalized, dynamic, and sensitive to perturbations [14, 16, 17]. It can thus be described as a delicate ecosystem and vaccination as a possible disruption of its homeostasis [18, 19]. However, the effect of PCVs, especially of PCV13, on other bacterial species that inhabit the same niche as *S. pneumoniae*, has not been extensively studied, and in particular, data of longitudinal analyses are still scarce [20]. The higher serotype coverage of PCV13 might, for example, have additional effects on the microbiota composition as compared to PCV7. This is, important, as the changes of the upper airway microbiota caused by the PCVs could potentially affect the susceptibility towards infectious diseases.

As for the methodology, sequencing of distinct variable regions of the 16S *rRNA* with subsequent definitions of operational taxonomic units (OTUs) based on 97% sequence identity has become a standard for the characterization of the microbiota [14, 17, 21]. As an alternative, oligotyping has been described which is able to detect very subtle variations among 16S *rRNA* sequences [22, 23]. However, no variable region of the 16S *rRNA* is appropriate for the pneumococcal species identification [24] and, therefore, *lytA* real-time PCR has been recommended for this purpose [25].

Within this study, we investigated the impact of vaccination with PCV7 or PCV13 on the pneumococcal carriage and the nasal microbiota in a case–control study using samples from a longitudinal cohort study of healthy infants in Switzerland. Our aims were (1) to assess pneumococcal carriage via quantitative PCR of the *lytA* gene, (2) to investigate changes in the nasal bacterial core microbiota composition using oligotyping, and (3) to compare the dissimilarity and stability of the bacterial core microbiota between the PCV7 and the PCV13 vaccine era.

## Methods

### Study design and nasal swab procedures

A total of 41 healthy infants, who received two doses of either PCV7 or PCV13 within the first year of life, were enrolled from the Basel Bern Infant Lung Development (BILD) cohort study [26]. Twenty infants were vaccinated with PCV7 and 21 with PCV13. No matching between the two groups was performed. These 41 infants were part of a previous study, where the nasal microbiota composition of 48 healthy infants was longitudinally investigated within the first year of life [16]. In the study here, seven infants were excluded because they obtained no or only a single dose of PCV. Pregnant mothers were recruited from the four major maternity hospitals, and practices of obstetricians in the agglomeration of Bern, Switzerland, and infants were followed weekly within the first year of life. As described in our previous study, information about pre-, peri-, and postnatal history was collected in hospital records and in a questionnaire [16]. In weekly phone interviews, study nurses assessed the infant’s health status, respiratory symptoms, and antibiotic prescriptions. Every second week, a nasal swab (nasal swabs and UTM tubes from Verridial E. Muller, Blonay, Switzerland) was collected by the parents of the study infants and sent in a transport medium within <10 days to the study center and frozen at −80 °C until further processing. The study parents were instructed by study nurses about correct and standardized sampling of the swabs. Like in our previous study, nasal swabs collected during antibiotic therapy were excluded from the study [16]. The study was approved by the ethics committee of the Canton of Bern, Switzerland.

### Quantitative real-time PCR of the pneumococcal *lytA* gene

DNA was extracted directly out of the swab (QIAamp DNA Minikit, Qiagen, Hilden, Germany) using 200 μl of transport medium. Quantitative real-time PCR (qPCR) of the pneumococcal-specific *lytA* gene (encoding for the LytA autolysin virulence factor [27]) was performed as described by Carvalho et al. [25]. We used the *lytA*-CDC primers and probe. The probe was labeled at the 5′-end with 6-carboxyfluorescin and the Black Hole Quencher 1 (BHQ1) was placed at the 3′-end of the probe. The assay was performed as a 20 μl reaction using the TaqMan Universal Master Mix (Applied Biosystems, Foster City, CA) according to the instructions of the manufacturer. The final concentration for the primers and the probe was 200 nM, and 2 μl of sample DNA was used. The experiment was carried out on the QuantStudio 7 Flex Real-Time PCR System using 0.1 ml MicroAmp Fast 96-Well Reaction Plates (Applied Biosystems). In each run, a standard curve was included, using serial tenfold dilutions (equivalent to 10,000,000—10 copies of genomic DNA of the strain *S. pneumoniae* 110.58, which was previously used in our lab [28–30]; accession number CP007593). A negative control was included in every run. The following cycling conditions were used: 50 °C for 2 min and 95 °C for 10 min, followed by 40 cycles of 95 °C for 15 s and 60 °C for 1 min. The results were collected using the QuantStudio 6 and 7 Flex Software. Each sample was measured in triplicate. We analyzed both, the *lytA* quantity (number of copies) and the number of samples positive for pneumococcal carriage (samples with >10 *lytA* copies). The *lytA* quantity was logarithmically transformed for analysis.

### *16S rRNA* sequencing

Following DNA extraction (see above), PCR of the V3–V5 region of the *16S rRNA* gene was performed using the primer pair 341F/907R. Primer sequences were as follows: 341F 5′-*CGTATCGCCTCCCTCGCGCCA*TCAGXXXXXXXXXX**ACTCCTACGGGAGGCAGCAG**-3′, and 907R 5′-*CTATGCGCCTTGCCAGCCCGC*TCAGXXXXXXXXXX**CCGTCAATTCMTTTGAGTTT**-3′ where the adaptor sequences are italicized, the template-specific sequences are in bold, and the XXXXXXXXXX sequences describe the sample-specific multiplex-identifier barcode. Primers were used in a final concentration of 100 μM. PCR was performed as a reaction mix, which included among the primers and the extracted DNA, FastStart Taq DNA Polymerase (1 U final), PCR Reaction Buffer without MgCl_2_ (10 μM final), MgCl_2_ (8 μM final), and dNTPs (0.2 mM final) (all from Roche Diagnostics, Mannheim, Germany). The PCR conditions were set to an initial denaturation and enzyme activation step of 95 °C for 6 min, followed by 35 cycles of 95 °C for 0.5 min, 59 °C for 0.5 min, 72 °C for 1.5 min, and a final elongation of 72 °C for 10 min. PCR products were cleaned up (Wizard SV Gel and PCR Clean-Up System, Promega, Madison, WI) and eluted in 40 μl of double-distilled H_2_O. Concentration of the PCR products was measured by the Agilent 2100 Bioanalyzer (Agilent Technologies, Basel, Switzerland). Sequencing was performed on the 454 GS FLX Titanium platform (Roche, Basel, Switzerland). Sequence reads were analyzed using the PyroTagger pipeline, which comprised quality filtering, removing of chimeras, and taxonomic assignment [31]. Pipeline settings were described in detail before [14]. In brief, singleton reads were first excluded and the read length was set to 230 nucleotides because a loss of quality scores was observed for subsequent nucleotides (data not shown). The built-in quality filtering steps of PyroTagger included for example the exclusion of reads in which 3% of bases had a Phred value <27, or the assumption that high-abundance reads are more accurate and therefore have a higher priority to be selected as the representative sequence. Representative sequences were then classified using the Phylodb, which contains 105,060 reference sequences from the SILVA (www.arb-silva.de) and greengenes (http://greengenes.lbl.gov) databases. Additional quality controls were as follows: Samples with a PCR product <1.0 ng/μl, corresponding to <1 pg/μl bacterial DNA [16], which was recommended as the threshold when working with low-density samples [32], were excluded, as well as samples with <70 sequence reads, and samples with >5.0% relative abundance in common with OTUs from two negative control samples. The reads were submitted to the National Center for Biotechnology Information Sequence Read Archive (accession number SRP041616).

### Oligotyping

We performed oligotyping of the core microbiota members, which were defined as the five most abundant bacterial families (*Moraxellaceae*, *Streptococcaceae*, *Staphylococcaceae*, *Corynebacteriaceae*, and *Pasteurellaceae*) [16]. Oligotyping allows elucidating the diversity within bacterial families based on the information that stems from the entropy analysis of variable sites in the sequence reads [23]. Sequence reads of every core microbiota family were selected and aligned using mothur version 1.36.1 [33]. Note that every core microbiota family was separately aligned (using the *align.seqs* command and the SILVA database) and sequence reads trimmed to the same length within the microbiota family. Read length depended therefore on the alignment of the microbiota family in the alignment space and differed between the families. The aligned sequence reads for each family were then subjected to the *otu2ot* pipeline [34], which was implemented in the R language [35], based on previous work by Eren et al. [23]. The cutoff was set to an entropy minimum of 0.6 and a minimal number of 21 sequence reads for each oligotype (OT). The “broken-stick model” was used to differentiate between real OTs and OTs that were probably due to chance alone [34]. The representative sequence of each OT, which was defined as the most abundant unique sequence belonging to an OT [22], was taxonomically assigned using BLAST (Basic Local Alignment Search Tool, http://blast.ncbi.nlm.nih.gov/Blast.cgi). The assignment/s with the highest “Max Score” was/were selected. The relative abundance of each OT was calculated as the fraction of reads of the total number of sequence reads in the corresponding sample.

### Microbiota analyses

Species richness, the Shannon Diversity Index (SDI), and the beta diversity as measured by the Jaccard dissimilarity index with non-metric multidimensional scaling (NMDS) as ordination method, were calculated in R using the *vegan* package as described before [16, 36]. Note that even though the Jaccard index is by definition binary based, vegan calculates both the binary-, and the abundance-based distances under the same name. We therefore performed both the binary- and the abundance-based dissimilarities using the same index. The richness was calculated using the *specnumber*, the SDI the *diversity*, and the beta diversity the *vegdist* and the *metaMDS* function. We used the relative abundance and the prevalence of the different OTs instead of the commonly used operational taxonomic units (OTUs) table as input matrix for the microbiota analyses. Clustering was performed based on the OT matrix using the Jensen–Shannon distance measure (as described by Arumugam et al. [37]) and the function *hclust* (*ward.D* distance) from the *stats* package in R. The optimal number of clusters was calculated via partitioning around medoids using the *pamk* function from the *fpc* package in R. Cluster correlation with either the PCV7 or the PCV13 era was evaluated with the function *rcorr* from the *Hmisc* package in R. Benjamini-Hochberg (BH) correction for multiple testing was additionally applied. The within-subject Jaccard dissimilarity by age was performed based on the OT table as input matrix and includes all pairwise comparisons from an infant within a 2-week interval in the corresponding month of age within the first year of life. We calculated both the abundance- and the binary-based Jaccard dissimilarity by age. In order to investigate the switch of an infant between clusters as a measure of community stability, the Jensen–Shannon distance was calculated using the cluster analysis as input as described by Gajer et al. [38]. The mean of all distance values per infant was calculated and logarithmically transformed. The lower the distance, the less the infant changed between community clusters.

### Statistical analyses

Risk and confounding factors, such as the pre-, peri-, and postnatal history, as well as respiratory symptoms, were compared between PCV7- and PCV13-vaccinated infants using chi-square tests and unpaired *t* tests. A negative binomial regression (NBR) model and two different linear mixed effect (LME) models were performed in R using the *glmmADMB* and the *lme4* package with the *glmmadmb* and the *lmer* function, respectively. We investigated the association between the vaccine era and the different outcome variables: pneumococcal *lytA* quantity and number of samples positive, SDI, richness, relative abundance and prevalence of OTs, and within-subject Jaccard dissimilarity. Based on the risk and confounding factors, we adjusted for season and age, which both were shown to impact the microbiota [16], as well as for the season of birth and symptoms of lower respiratory tract infection (LRTI) [26]. To investigate our first and second aim—the analysis of the pneumococcal carriage and the nasal core microbiota—models 1 and 2 were performed: Model 1 was used for the following outcome variables: *lytA* quantity and number of samples positive, OT prevalence based on the binary matrix, SDI, and richness. The *lytA* quantity corresponds to the total number of *lytA* copies, while the number of samples positive represents the number of samples with >10 *lytA* copies. The fixed effects for model 1 were age, season, season of birth, symptoms of LRTI, and vaccine era. Because the outcome variable relative abundance of OTs was not normally distributed, we used a negative binomial regression (NBR) model as suggested before [39]. Model 2 was therefore used for the relative abundance of the OTs as outcome variable. Same as for model 1 the fixed effects were age, season, season of birth, symptoms of LRTI, and vaccine era. To investigate our third aim—the dissimilarity and stability of the core microbiota—model 3 was performed for the Jaccard dissimilarity by age as outcome variable. We therefore stratified the dissimilarity to the corresponding month of age. Because the Jaccard dissimilarity is based on pairwise comparisons between two samples at two different time points, the variable does not allow to unambiguously defining the season for each value. We therefore decided to omit this fixed effect. The fixed effects were: age, season of birth, and vaccine era. As random effects we entered for all three models intercepts for the infants, as well as by-infant random slopes for the effect of the vaccine era. The difference of the mean Jensen–Shannon distance between the PCV7 and the PCV13 era was calculated by a *t* test. The microbiome based sum of powered score tests (MiSPU; R package and command *MiSPU*) was performed to investigate the effect of the vaccine era on the bacterial microbiota composition [40]. The MiSPU was performed using the OT abundance-based matrix as input and age, season, the season of birth, symptoms of LRTI, and the infant identifier as covariates. The phylogenetic tree of the OT matrix was rooted at the group of “others.” Graphical representations and statistical analyses were either performed in R version 3.3.0 or in GraphPad Prism version 7.01 (GraphPad Software, La Jolla, CA).

## Results

### Study cohort and samples

This study included 41 infants, who received two doses of PCV within the first year of life (Table 1). Samples were collected between April 2010 and December 2013. After exclusion of low-quality samples and samples obtained during antibiotic therapy, a total of 763 samples were included. This corresponds to a mean (±standard deviation (SD)) of 18.6 (±3.3) samples per infant. In our study cohort, 20 infants were vaccinated with PCV7 (*n* = 355 samples) and 21 infants with PCV13 (*n* = 408 samples). The recommended vaccination schedule for PCV in Switzerland is at 2, 4, and 11–15 months of age. Note that none of the infants included here received the third dose of PCV before the 12th month of age. The mean age (±SD) at the first and the second dose was 10.1 (±3.6) and 18.9 (±4.1) weeks, respectively (Table 1). We compared different risk and confounding factors between PCV7- and PCV13-vaccinated infants and found that the season of birth and symptoms of LRTI differed significantly between the two groups (Table 1; 2 × 4 chi-square test and unpaired *t* test; *P* = 0.03 and *P* = 0.05, respectively). We subsequently added these two potentially confounding factors to our LME and NBR models.

**Table 1:** Characteristics of the study population and comparison of risk and confounding factors between PCV7- and PCV13-vaccinated infants

Characteristic	PCV7	PCV13	*P* value
Number of infants, *n* (%)	20 (48.8%)	21 (51.2%)	
Gender (male), *n* (%)	10 (50%)	9 (42.9%)	0.76^#^
Season of birth, *n* (%)			*0.03* ^#^
Winter	2 (10%)	8 (38.1%)	
Spring	4 (20%)	8 (38.1%)	
Summer	7 (35%)	3 (14.3%)	
Fall	7 (35%)	2 (9.5%)	
^a^Parental education, *n* (%)			0.51^#^
Low	2 (10%)	4 (19.1%)	
Middle	6 (30%)	8 (38.1%)	
High	12 (60%)	9 (42.9%)	
^b^HA nutrition, *n* (%)	4 (20%)	4 (19.1%)	0.94^#^
C-section, *n* (%)	3 (15%)	3 (14.3%)	0.95^#^
^c^Childcare, *n* (%)	5 (25%)	5 (23.8%)	0.93^#^
Smoking during pregnancy, *n* (%)	2 (10%)	1 (4.8%)	0.52^#^
^d^Smoking exposure in the 1st year, *n* (%)	2 (10%)	5 (23.8%)	0.24^#^
^e^Maternal atopy, *n* (%)	6 (30%)	4 (19.1%)	0.41^#^
Siblings, *n* (%)			0.18^#^
0	3 (15%)	5 (23.8%)	
1	9 (45%)	13 (61.9%)	
≥2	8 (40%)	3 (14.3%)	
Gestational age at birth [weeks], mean (±SD)	39.8 (±1.2)	39.4 (±2.1)	0.45^$^
Length at birth [cm], mean (±SD)	49.6 (±1.7)	49.7 (±2.0)	0.78^$^
Weight at birth [g], mean (±SD)	3403.5 (±334.2)	3343.3 (±581.2)	0.69^$^
Breastfeeding duration [months], mean (±SD)	8.6 (±2.7)	9.3 (±2.9)	0.44^$^
^f^Age at PCV administration [weeks], mean (±SD)			
1^st^ dose	9.6 (±1.8)	10.6 (±4.8)	0.34^$^
2^nd^ dose	18.9 (±2.4)	18.9 (±5.5)	0.97^$^
^g^Hib administration, *n*, mean age [weeks] (±SD)			
1^st^ dose	20, 9.6 (±1.8)	21, 9.6 (±1.1)	0.96^$^
2^nd^ dose	20, 18.9 (±2.4)	21, 18.2 (±1.1)	0.28^$^
3^rd^ dose	18, 27.1 (±1.9)	21, 27.2 (±1.6)	0.87^$^
Respiratory symptoms, *n*, mean per infant (±SD)			
Total	127, 6.4 (±2.7)	134, 6.4 (±3.1)	0.97^$^
Rhinitis	99, 5.0 (±2.4)	125, 6.0 (±2.9)	0.24^$^
^h^URTI	71, 3.6 (±2.2)	76, 3.6 (±2.4)	0.92^$^
^i^LRTI	7, 0.35 (±0.8)	21, 1.0 (±1.2)	*0.05* ^$^
Wheezing	2, 0.1 (±0.3)	6, 0.3 (±0.7)	0.29^$^

Statistically significant differences were indicated in italics

*SD* standard deviation

^a^Parental education was categorized into low (less than 4 years of apprenticeship), middle (at least 4 years of apprenticeship), or high (tertiary education)

^b^HA nutrition (hypoallergenic nutrition) was defined as feeding of hypoallergenic milk supplements at any time point within the first year of life

^c^Childcare was defined as attending childcare at any time point within the first year of life

^d^Smoking exposure due to the father and/or the mother smoking within the first year of life of the infant

^e^Maternal atopy was defined as asthma, hay fever, or eczema

^f^The PCV (pneumococcal conjugate vaccine) vaccination schedule is at 2, 4, and 11–15 months Note that all infants obtained the first and the second but not the third dose within the first year of life

^g^Hib (*Haemophilus influenzae* type b) vaccination schedule is at 2, 4, 6, and 15–24 months of age. None of the infants got the fourth dose within the observed study period. In Switzerland, the vaccine is recommended as a combination vaccine with diphtheria, tetanus, pertussis, and poliomyelitis

^h^URTI: Symptoms of upper respiratory tract infection (URTI) was defined as typical upper respiratory tract symptoms, whereas cough and/or wheeze had to be present

^i^LRTI: Symptoms of lower respiratory tract infection (LRTI) was defined as cough, wheeze or breathing difficulties, combined with upper respiratory tract symptoms or elevated body temperature for more than two consecutive days

^#^Statistical testing of categorical variables was performed by a 2 × 2, 2 × 3, or 2 × 4 chi-square test

^$^Statistical testing of continuous variables was performed by unpaired *t* tests

### Lower pneumococcal carriage in the PCV13 era

**Fig. 1:**
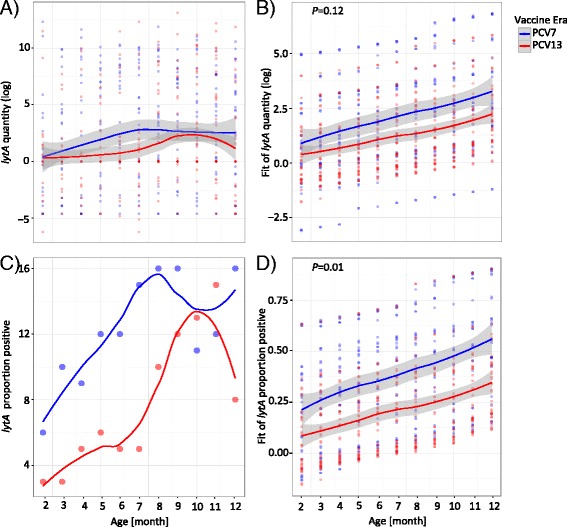
Pneumococcal carriage. Samples from the PCV7 era (*blue*, *n* = 355) were compared to samples from the PCV13 era (*red*, *n* = 408). Shown are the raw data (**a**, **c**) and the fitted data (**b**, **d**) using linear mixed effect (LME) models. The fixed effects were age, season, season of birth, symptoms of lower respiratory tract infection (LRTI), and vaccine era. The random effects were intercepts for the infants and by-infant random slopes for the effect of the vaccine era. Lines are based on a local polynomial regression fitting (*loess* function in R). *Gray bands* indicate the standard deviation (SD). **a** Pneumococcal carriage as measured by the *lytA* quantity (log transformed). **b** Fitted values of the *lytA* quantity using the LME model. There was no significant difference between the PCV7 and the PCV13 era (LME model; *P* = 0.12). **c** Number of samples positive for pneumococcal carriage as defined by >10 *lytA* copies. **d** Fitted values of the number of samples positive using the LME model. Significantly lower number of samples positive for *lytA* in the PCV13 as compared to the PCV7 era (LME model; *P* = 0.01)

We performed qPCR of the pneumococcal *lytA* gene to analyze pneumococcal carriage within the first year of life. When the data was analyzed quantitatively, there was no significant difference in pneumococcal carriage between the PCV7 and the PCV13 era (Fig. 1a, b). However, when the number of samples positive for pneumococcal carriage (>10 *lytA* copies) was analyzed, we found, in contrast, a significantly lower carriage in the PCV13 era (Fig. 1c, d and Additional file 1: Table S1; LME model; *P* = 0.01). This was also true using a univariate LME (data not shown). These results indicate a decreased pneumococcal carriage in the PCV13 era. We therefore hypothesized that the different pneumococcal colonization in the PCV13 era could lead to a change in the microbiota profile.

### Oligotyping of the bacterial Core microbiota

**Fig. 2:**
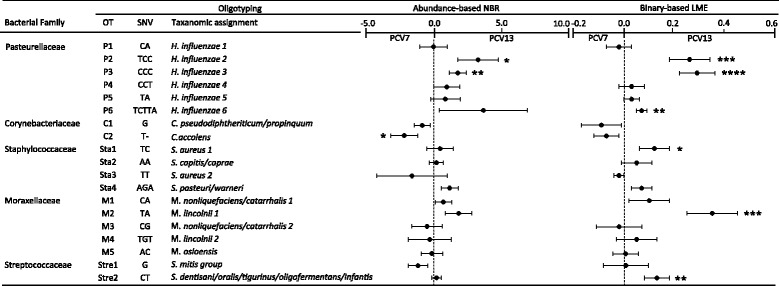
Oligotyping of the bacterial core microbiota. Indicated are the bacterial families of the core microbiota and the corresponding oligotypes (OTs), single-nucleotide polymorphisms (SNPs), and the taxanomic assignment. The latter was derived by choosing a representative sequence of each OT, which was defined as the most abundant unique sequence belonging to an OT, and assigning the taxanomy by using BLAST (Basic Local Alignment Search Tool). A negative binomial regression (NBR) model was used for the relative abundance of the OTs and a linear mixed effect (LME) model for the binary-based analysis. The fixed effects of both models were age, season, season of birth, symptoms of lower respiratory tract infection (LRTI), and vaccine era. The random effects were intercepts for the infants and by-infant random slopes for the effect of the vaccine era. The PCV7 era (*n* = 355 samples) was compared to the PCV13 era (*n* = 408 samples), whereas the PCV7 era was used as baseline. Inputs were either the relative abundance of the OTs (abundance-based NBR model) or the binary matrix (binary-based LME model). Indicated are the Estimates and the Standard Errors. Abundance-based NBR: OT P2 (*H. influenzae 2*) and P3 (*H. influenzae 2*) significantly increased and OT C2 (*C. accolens*) decreased in the PCV13 era as compared to the PCV7 era (*P* = 0.03, *P* = 0.005, and *P* = 0.04, respectively). Binary-based LME: following OTs were significantly increased in the PCV13 era: P2 (*H. influenzae 2*), P3 (*H. influenzae 3*), P6 (*H. influenzae 6*), Sta1 (*S. aureus 1*), M2 (*M. lincolnii 2*), and Stre2 (*S. dentisani/oralis/tigurinus/oligofermentans/infantis*) (*P* = 0.001, *P* = 0.0001, *P* = 0.002, *P* = 0.04, *P* = 0.0004, and *P* = 0.003, respectively)

We investigated the impact of the PCV13 and the PCV7 era on the nasal microbiota based on *16S rRNA* sequencing. The 763 samples analyzed for this study produced a mean number (±SD) of 1227.3 sequence reads (±924.4) per sample. We selected the reads of the five most abundant bacterial families (*Moraxellaceae*, *Streptococcaceae*, *Staphylococcaceae*, *Pasteurellaceae*, and *Corynebacteriaceae*), which were defined as the core microbiota, and performed oligotyping. Oligotyping results in a more precise taxonomic assignment as compared to the OTU output as it relies on distinct single-nucleotide polymorphisms (SNPs). In total, we identified 19 oligotypes (OTs) (Fig. 2 and Additional file 1: Table S2) and exact SNP position due to *E. coli* numbering was received (Additional file 1: Table S2). Within the bacterial family of *Pasteurellaceae*, we found six oligotypes which were taxonomically assigned as *Haemophilus influenzae*. The *Staphylococcaceae* OTs were differentiated into *Staphylococcus aureus* (OTs Sta1 and Sta3) and coagulase-negative *Staphylococci* (OTs Sta2 and Sta4). However, oligotyping was not able to differentiate between *Moraxella catarrhalis* and *Moraxella nonliquefaciens*, as well as it was not able to differentiate between *Streptococcus pneumoniae* and *Streptococcus pseudopneumoniae*. While for the differentiation of *Moraxella* spp. the analysis of V1–V3 is likely to be superior (data not shown), it is known that the whole 16S rRNA gene is not able to clearly identify S. pneumoniae which is, in part, due to conservation of the 16S rRNA sequence within the Mitis group of streptococci [24]. We next compared the relative abundance of OTs between the PCV7 and the PCV13 era. The relative abundance of the OTs was not normally distributed; we therefore used a NBR model for this analysis. We found OTs P2 (*H. influenzae 2*) and P3 (*H. influenzae 3*) significantly increased in the PCV13 era, but OT C2 (*Corynebacterium accolens*) decreased as compared to the PCV7 era (Fig. 2 and Additional file 1: Table S2; NBR model; *P* = 0.03, *P* = 0.005, and *P* = 0.04, respectively). The later, however, was not significant after correction for multiple testing with Benjamini-Hochberg (BH). In order to account for the low-abundant OTs we repeated the analysis using the binary-based matrix of the OTs and the LME model for analysis (Fig. 2 and Additional file 1: Table S2). In contrast to the abundance-based output, the binary-based analysis resulted in several different significantly increased OTs in the PCV13 era (Fig. 2 and Additional file 1: Table S2; NBR model; OTs P2 (*H. influenzae 2*), P3 (*H. influenzae 3*), P6 (*H. influenzae 6*), Sta1 (*S. aureus*), M2 (*Moraxella lincolnii*), Stre2 (*S. dentisani/oralis/tigurinus/oligofermentans/infantis*); *P* = 0.001, *P* = 0.0001, *P* = 0.002, *P* = 0.04; *P* = 0.0004, *P* = 0.003, respectively).

### Increased bacterial diversity in the PCV13 era

**Fig. 3:**
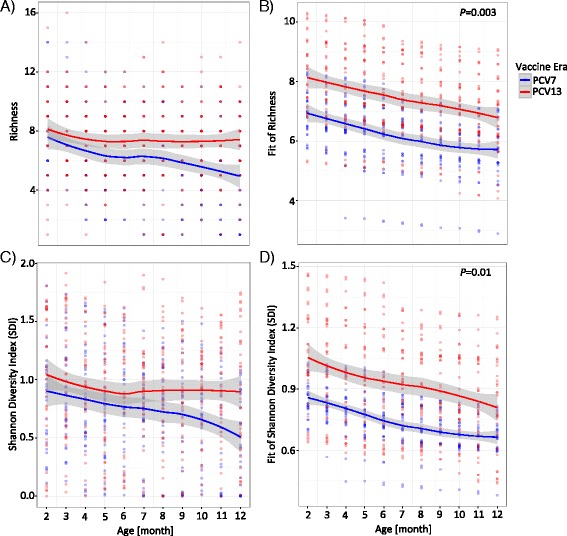
Alpha diversity of the bacterial core microbiota. Samples from the PCV7 era (*blue*, *n* = 355) were compared to samples from the PCV13 era (*red*, *n* = 408). Shown are the raw data (**a**, **c**) and the fitted data (**b**, **d**) using linear mixed effect (LME) models. The fixed effects were age, season, season of birth, symptoms of lower respiratory tract infection (LRTI), and vaccine era. The random effects were intercepts for the infants and by-infant random slopes for the effect of the vaccine era. Lines are based on a local polynomial regression fitting (*loess* function in R). *Gray bands* indicate the standard deviation (SD). Inputs are based on the relative abundance or the prevalence of oligotypes (OT). **a** OT richness as calculated by the binary-based matrix. **b** Fitted values of the OT richness. Significantly higher richness in the PCV13 era as compared to the PCV7 era (LME model; *P* = 0.003). **c** Shannon Diversity Index (SDI) as measured by the relative abundance-based input. **d** Significantly higher SDI in the PCV13 era as compared to the PCV7 era (LME model; *P* = 0.01)

The increased pneumococcal carriage in samples from PCV7-vaccinated infants let us further hypothesize a decreased alpha diversity (within-sample diversity) in the PCV7 era, due to a potential dominance of a bacterial community member. Based on the binary-based input matrix, we calculated the number of OTs (richness) and found that samples from the PCV13 era had, indeed, a constantly significantly higher richness within the first year of life (Fig. 3a, b and Additional file 1: Table S1; LME model; *P* = 0.003). We also calculated the Shannon Diversity Index (SDI) based on the relative abundance of the OTs and found that the SDI was significantly increased in the PCV13 era, too (Fig. 3c, d and Additional file 1: Table S1; LME model; *P* = 0.01). This was also true using a univariate LME (data not shown).

**Fig. 4:**
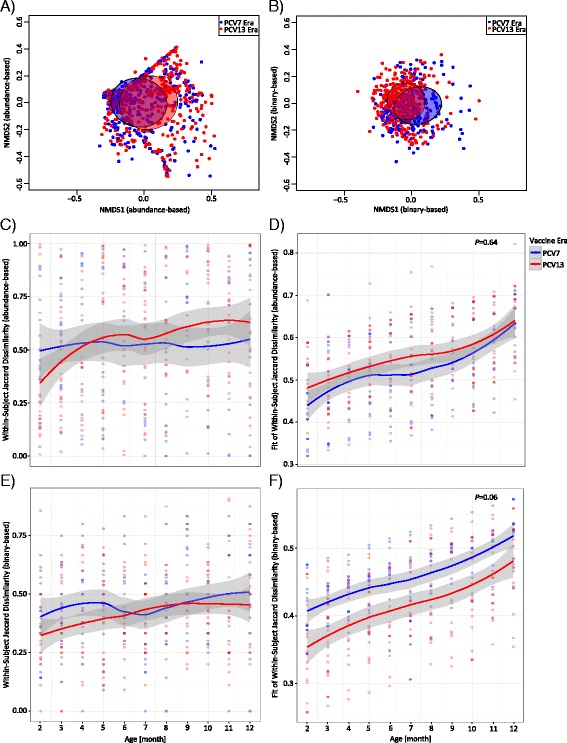
Beta diversity of the bacterial core microbiota. Samples from the PCV7 era (*blue*, *n* = 355) were compared to samples from the PCV13 era (*red*, *n* = 408). Non-metric multidimensional scaling (NMDS) was performed based on the Jaccard dissimilarity matrix of **a** the abundance-based and **b** the binary-based oligotype (OT) input. Within-subject Jaccard dissimilarity was longitudinally analyzed by age. Shown are the raw data (**c**, **e**) and the fitted data (**d**, **f**) using linear mixed effect (LME) models. The fixed effects were age, season of birth, and vaccine era. The random effects were intercepts for the infants and by-infant random slopes for the effect of the vaccine era. Lines are based on a local polynomial regression fitting (*loess* function in R). Gray bands indicate the standard deviation (SD). **c** Abundance-based within-subject Jaccard dissimilarity within the first year of life. **d** Fitted values of the abundance-based within-subject Jaccard dissimilarity. There was no significantly different dissimilarity between the PCV7 and the PCV13 era (LME model; *P* = 0.64). **e** Binary-based within-subject Jaccard dissimilarity within the first year of life. **f** Fitted values of the binary-based within-subject Jaccard dissimilarity. There was a trend of an increased dissimilarity of the PCV7 as compared to the PCV13 era using the LME model (LME model; *P* = 0.06)

We next investigated the beta diversity (between-sample diversity) of samples from the PCV7 and the PCV13 era and performed abundance- and binary-based NMDS analyses (Fig. 4a, b. We used the multivariate testing method MiSPU (microbiome-based sum of powered score tests correcting for the covariates age, season, symptoms of LRTI, the season of birth, and the infant identifier) to calculate the difference between the overall microbiota composition in the PCV7 and the PCV13 era. The test revealed that the vaccine era had an effect on the microbiota composition, which was, however, not statistically significant (MiSPU; *P* = 0.06). We then looked at the Jaccard dissimilarity of each study infant longitudinally within the first year of life. We found no difference of the abundance-based Jaccard dissimilarity by age using the LME model (Fig. 4c, d and Additional file 1: Table S1). There was, however, a trend of a decreased Jaccard dissimilarity in the PCV13 as compared to the PCV7 era using the binary-based input matrix (Fig. 4e, f and Additional file 1: Table S1; LME model; *P* = 0.06). These results indicate a higher stability of the microbiota composition over time in samples of PCV13- as compared to PCV7-vaccinated infants.

### Increased cluster diversity and stability in the PCV13 era

**Fig. 5:**
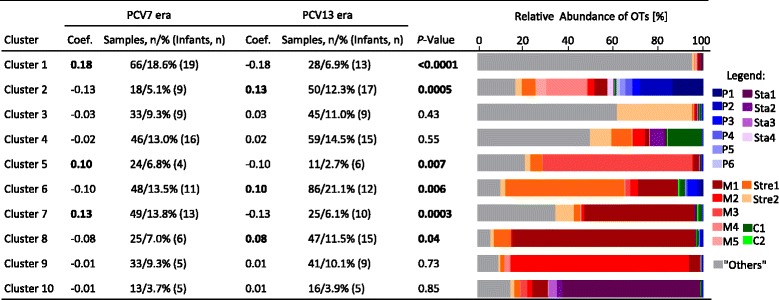
Cluster analysis. Indicated are the clusters, the correlation coefficients of the PCV7 (*n* = 355 samples and *n* = 20 infants) and the PCV13 era (*n* = 408 samples and *n* = 21 infants), and the *P* values. On the right, the composition of the clusters based on the oligotypes (mean relative abundances) is shown. A positive correlation was found for clusters 1, 5, and 7 with the PCV7 era (coefficients 0.18, 0.10, and 0.13; *P* < 0.0001, *P* = 0.007, and *P* = 0.0003, respectively), and for clusters 2, 6, and 8 with the PCV13 era (coefficients 0.13, 0.10, and 0.08; *P* = 0.0005, *P* = 0.006 and *P* = 0.04, respectively). Significantly positive correlations are indicated in *bold*

We next performed hierarchical clustering based on the relative abundance of the OTs. We obtained 10 different clusters, which were each dominated by at least one OT (Fig. 5). Correlation analysis revealed a positive correlation of clusters 1, 5, and 7 with the PCV7 era (Fig. 5; coefficient 0.18, 0.10, and 0.13; *P* < 0.0001, *P* = 0.007, and *P* = 0.0003, respectively). Regarding the PCV13 era, we found a positive correlation with clusters 2, 6, and 8 (Fig. 5, coefficients 0.13, 0.10, and 0.08; *P* = 0.0005, *P* = 0.006 and *P* = 0.04, respectively). We calculated the SDI of each cluster and found that the PCV13-associated cluster 2 had the highest SDI among the ten different clusters. In contrast, the PCV7-associated clusters were among those with the lowest SDI (Additional file 1: Table S3).

**Fig. 6:**
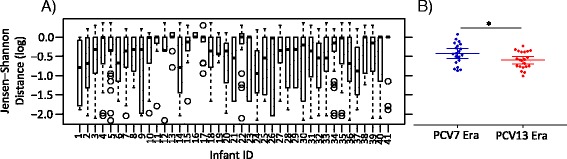
Cluster stability of the bacterial core microbiota. Cluster stability was calculated by the Jensen–Shannon distance based on the cluster matrix as input. A lower Jensen–Shannon distance indicates a higher stability because fewer switches between clusters occur. **a** Indicated is the within-subject Jensen–Shannon distance for each infant. Infant numbers 1–20 were vaccinated with the PCV7 and infant numbers 21–41 with the PCV13. **b** The mean within-subject Jensen–Shannon distance between PCV7-vaccinated infants (*n* = 20) and PCV13-vaccinated infants (*n* = 21) was compared. There was a significantly lower mean Jensen–Shannon distance in the PCV13 era as compared to the PCV7 era (*t* test; *P* = 0.03).

Defining a microbiota with a lower diversity and a higher dissimilarity as potentially instable [41], we hypothesized a decreased cluster stability of bacterial communities in the PCV7 as compared to the PCV13 era. Thus, we calculated the Jensen–Shannon distances based on the cluster matrix as input. By definition, communities that remain in the same cluster over time display a high level of stability, while those often switch between clusters have low levels of stability [38]. We found that infants vaccinated in the PCV13 era changed less between the clusters and thus had a significantly lower mean Jensen–Shannon distance as compared to infants vaccinated in the PCV7 era (Fig. 6a, b, Additional file 1: Table S1, and Additional file 2; *t* test; *P* = 0.03).

## Discussion

Here, we performed a case–control study with samples from a longitudinal infant cohort, investigating the effect of PCV7 as compared to PCV13 on pneumococcal carriage and the nasal microbiota composition in healthy infants within the first year of life in Switzerland. The density of pneumococcal carriage remained unchanged, but in contrast, the number of samples positive for *S. pneumoniae* was decreased in the PCV13 era, which could indicate a higher effectiveness of the PCV13 as compared to the PCV7 in reducing carriage. Furthermore, we found significant differences in the microbiota composition between infants vaccinated in the PCV7 and the PCV13 era. Samples from the PCV13 era were characterized by an increased sample diversity and richness as compared to samples from the PCV7 era. In accordance, the PCV13 era was associated with clusters of a high diversity. In addition, the within-subject microbiota dissimilarity (based on the binary distance matrix) and the Jensen–Shannon distance was decreased in infants born in the PCV13 era, indicating higher microbiota stability in the PCV13 era.

Infection with *S. pneumoniae* starts with upper airway colonization, which might progress to invasive disease if immunological barriers are crossed. In order to protect from invasive pneumococcal disease, colonization needs therefore to be prevented [2, 3]. With the introduction of the PCVs, pneumococcal serotype replacement and a decrease of pneumococcal carriage has been described [42, 43]. In our study, *16S rRNA* sequencing and subsequent oligotyping did not allow to reliably differentiate between *S. pneumoniae* and other members of the *Streptococcus mitis* group, such as *S. pseudopneumoniae*. This problem has been previously acknowledged [44], and strategies how to tackle this issue have been addressed [45]. In our study, we performed qPCR of the *lytA* gene, which was shown to be highly specific for *S. pneumoniae* [25, 46–48]. We found that the presence of *S. pneumoniae* was lower in the PCV13 as compared to the PCV7 era. In contrast, the microbiota diversity was increased in samples from the PCV13 era. Together, these results are in line with previous studies, where the carriage rates of *S. pneumoniae*, but also the potential pathogens *M. catarrhalis* and *H. influenzae* have been associated with a lower diversity of the upper respiratory tract microbiota in children [21]. Furthermore, concerns have already been raised over the increased carriage of potential pathogens, such as *H. influenzae*, upon vaccination with PCV [49, 50]. In accordance with these culture-based studies, we found an increase of a specific *H. influenzae* (oligotype P2) in the PCV13 era. However, this increased abundance was not associated with a disordered microbiota or an increased risk of symptoms of upper respiratory tract infections (URTI) in the PCV13 era. It seems, in general, challenging to define specific bacterial genera to be predictive for an increased risk of respiratory infections: An Australian study found that transient incursions of *Streptococcus*, *Moraxella*, or *Haemophilus* marked virus-associated acute respiratory infections, while a Dutch study, in contrast, found fewer URTIs in *Moraxella*-dominated microbiota profiles [51, 52]. A recent prospective cohort study from the USA investigating the nasopharyngeal microbiota in healthy children aged 49–84 months, found that the time to develop an URTI was significantly positively correlated with the microbiota diversity, and children who experienced more frequent URTIs were found to harbor a microbiota of reduced diversity [53]. Even though we did not observe a difference in the prevalence of URTI symptoms between PCV7- and PCV13-vaccinated infants, the abovementioned studies suggest that the microbiota diversity, rather than specific bacterial genera, could serve as a marker to predict the susceptibility of infections.

In addition to the increased diversity, we identified increased microbiota stability in infants vaccinated with PCV13 as compared to PCV7. The higher microbiota stability might be a result of the increased diversity, in so far as a diverse microbiota could indicate the presence of commensal species rather than pathobionts, whereas the latter might be associated with a fluctuating and instable microbiota [21]. Only recently, commensal genera such as *Corynebacterium* and *Dolosigranulum* were associated with niche stability over time [54]. In similar manner, the gram-positive *Alloiococcus* was described as a stable component of the healthy nasopharyngeal microbiota, which was associated with an enhanced overall stability of the microbiota [51]. In addition to the increased diversity as discussed above, the higher stability of the microbiota in the PCV13 era could therefore indicate a lower susceptibility to infectious diseases of the upper airways because of the lower turnover of bacterial communities [38]. Indeed, lower incidence rates of acute otitis media have already been reported upon PCV13 but not as much upon PCV7 introduction [55, 56]. In our study here, we observed no difference in the number of symptoms from URTIs between PCV7- and PCV13-vaccinated infants. However, an overall reduction of acute otitis media cases in the PCV13 era in Switzerland has been observed [57]. Overall, this indicates that the lower carriage rate and the higher diversity could lead to a lower susceptibility of respiratory tract infections in PCV13- as compared to PCV7-vaccinated infants.

Our study has some major strengths: To our best knowledge, this is the first study comparing the nasal bacterial microbiota between PCV7- and PCV13-vaccinated infants using a longitudinal study design. Furthermore, we performed oligotyping, which improved the resolution of the taxonomic assignments in comparison to the standard taxon-based assignment [58]. This allowed us to identify some reliable changes of community members upon vaccination with the PCVs up to the species level. In addition, performing a longitudinal study allowed the investigation of the stability—a marker of bacterial turnover—of the microbiota. Therefore, this study could be used to assist in analyzing cross-sectional studies of the nasal microbiota in infants.

A limitation of this study is the absence of pre-vaccine era samples, which did not allow comparing the PCV7 and the PCV13 era to the era before the implementation of the PCVs. Therefore, we were only able to investigate the changes upon replacement of the PCV7 by the PCV13. However, different studies already showed a bigger impact of the PCV13 on the pneumococcal carriage as compared to PCV7 [59, 60]. This replacement clearly induced a change in the nasal microbiota and is thus of importance not only in Switzerland but also in other countries with similar vaccination schedules.

Furthermore, our observed changes cannot clarify if our findings are additionally influenced by herd protection due to early and/or late consequences of the PCV13 or PCV7 introduction, respectively. We only can say that we noted the changes in the PCV13 era. As for the decrease of pneumococcal carriage in the PCV13 era, this has also recently been described in a rather large cross-sectional study investigating pneumococcal carriage in patients with acute otitis media in Switzerland [57]. In combination with this study, this may indicate that non-PCV13 serotypes may not colonize equally well and, therefore, this may vacate the niche for other “colonizers.”

It has also to be noted that we did not analyze the entire microbiota with oligotyping but only the most abundant bacterial families. Therefore, a more in-depth inclusion of “others” may have led to different and/or additional conclusions. However, we hypothesized that the pneumococcal conjugate vaccines may only have subtle consequences on specific members of the bacterial families with the highest abundances (core microbiota) and, therefore, a more depth analysis of these families using oligotyping would be superior in this case as compared to the more general 97% OTU approach of the entire microbiota.

Another limitation of the study is the small sample size including only 41 infants. However, we provide an extreme dense longitudinal sampling which allows an in-depth analysis of the stability of the microbiota within the first year of life.

## Conclusions

This study shows changes in the nasal microbiota composition between infants vaccinated in the PCV7 and the PCV13 era. Importantly, samples from the PCV13 era were associated with a higher diversity and stability, which might be consistent with a lower susceptibility to respiratory tract infections. However, whether this hypothesis holds true, needs further investigations—not only by conventional culture studies but also by longitudinal, sequencing-based microbiota analyses.

## Additional files

Additional file 1: Table S1. Adjusted analysis of the association of different variables with the vaccine era using linear mixed effect models. **Table S2.** Oligotyping Output and Adjusted Analysis of the Association of Oligotype Abundances and the Vaccine Era Using Negative Binomial Regression and Linear Mixed Effect Models. **Table S3.** Shannon Diversity Index of Clusters. (DOCX 23 kb)

Additional file 2: Suplementary Figure. (PDF 1873 kb)

## Abbreviations

BLAST: Basic Local Alignment Search Tool
Coeff: Coefficient
HA nutrition: Hypoallergenic nutrition
Hib: *Haemophilus influenzae* type b vaccine
IPD: Invasive pneumococcal disease
LME: Linear mixed effect model
LRTI: Lower respiratory tract infection
MiSPU: Microbiome-based sum of powered scores test
NBR: Negative binomial regression model
NMDS: Non-metric multidimensional scaling
OT: Oligotype
OTU: Operational taxonomic unit
PCV: Pneumococcal conjugate vaccine
SD: Standard deviation
SDI: Shannon Diversity Index
SNP: Single-nucleotide polymorphism
URTI: Upper respiratory tract infection
