# Supplementary material for: Influence of the pneumococcal conjugate vaccines on the temporal variation of pneumococcal carriage and the nasal microbiota in healthy infants: a longitudinal analysis of a case–control study
Source: Microbiome. 2017 Jul 24;5:85. doi: 10.1186/s40168-017-0302-6 (PMC5525364; doi:10.1186/s40168-017-0302-6)
Supplement: Supplementary file 1 — Adjusted analysis of the association of different variables with the vaccine era using linear mixed effect models. Table S2. Oligotyping Output and Adjusted Analysis of the Association of Oligotype Abundances and the Vaccine Era Using Negative Binomial Regression and Linear Mixed Effect Models. Table S3. Shannon Diversity Index of Clusters. (DOCX 23 kb) [file 40168_2017_302_MOESM1_ESM.docx]

**Additional 1: Table S1. Adjusted Analysis of the Association of Different Variables with the Vaccine Era Using Linear Mixed Effect Models.**

| Variable | Estimate | Standard Error | *P*-value |
| --- | --- | --- | --- |
| Overall bacterial density | -10.52 | 4.51 | 0.02 |
| *lytA* quantity (log) | -1.10 | 0.71 | 0.12 |
| Number of samples positive for *lytA* | -0.21 | 0.08 | 0.01 |
| Richness | 1.36 | 0.46 | 0.003 |
| SDI | 0.19 | 0.07 | 0.01 |
| Jaccard dissimilarity (abundance-based) | -0.02 | 0.05 | 0.70 |
| Jaccard dissimilarity (binary-based) | -0.06 | 0.03 | 0.06 |

**Table S1**: Adjusted Analysis of the Association of Different Variables with the Vaccine Era Using Linear Mixed Effect Models. Outcome variables were: bacterial density (as measured by the *16S rRNA* PCR concentration), pneumococcal *lytA* quantity (log) and number of samples positive (>10 copies of *lytA*) as a measure for pneumococcal carriage, richness, Shannon Diversity Index (SDI), and within-subject Jaccard dissimilarity (abundance- and binary-based) by age. Baseline was the PCV7 vaccine era. PCV7 era: n=20 infants and n=355 samples, PCV13 era: n=21 infants and n=408 samples.

**Supplementary Table 2: Oligotyping Output and Adjusted Analysis of the Association of Oligotype Abundances and the Vaccine Era Using Negative Binomial Regression and Linear Mixed Effect Models.**

|  | Oligotyping | | | Abundance-based NBR model | | | | Binary-based LME model | | | |
| --- | --- | --- | --- | --- | --- | --- | --- | --- | --- | --- | --- |
| Bacterial Family | OT | SNV | *E. coli* Numbering for SNPs | Est. | Std.E | *P*-value | Est. | | Std.E | *P*-value |  |
| Pasteurellaceae | P1 | CA | C614C, C422A | -0.01 | 1.02 | 0.99 | -0.02 | | 0.05 | 0.69 |  |
|  | P2 | TCC | C614T, C422C, C470C | 3.31 | 1.49 | **0.03** | 0.26 | | 0.08 | **0.001** |  |
|  | P3 | CCC | C614C, C422C, C470C | 1.80 | 0.64 | **0.005** | 0.29 | | 0.07 | **0.0001** |  |
|  | P4 | CCT | C614C, C422C, C470T | 0.99 | 0.97 | 0.31 | 0.03 | | 0.05 | 0.57 |  |
|  | P5 | TA | C614T, C422A | 0.87 | 1.10 | 0.43 | 0.03 | | 0.03 | 0.28 |  |
|  | P6 | TCTTA | C614T, C422C, C470T, A456T, G626A | 3.70 | 3.29 | 0.26 | 0.07 | | 0.02 | **0.002** |  |
| Corynebacteriaceae | C1 | G | G455G | -0.85 | 0.60 | 0.16 | -0.09 | | 0.08 | 0.27 |  |
|  | C2 | T- | G455T, T472/473-* | -2.16 | 1.03 | **0.04** | -0.07 | | 0.05 | 0.11 |  |
| Staphylococcaceae | Sta1 | TC | G455T, C470C | 0.49 | 0.99 | 0.62 | 0.12 | | 0.06 | **0.04** |  |
|  | Sta2 | AA | G455A, T472A | 0.21 | 0.52 | 0.69 | 0.05 | | 0.06 | 0.43 |  |
|  | Sta3 | TT | G455T, C470T | -1.61 | 2.61 | 0.54 | -0.02 | | 0.02 | 0.46 |  |
|  | Sta4 | AGA | G455A, T472G, A478A | 1.19 | 0.63 | 0.06 | 0.07 | | 0.04 | 0.06 |  |
| Moraxellaceae | M1 | CA | C477C, A459A | 0.73 | 0.61 | 0.24 | 0.10 | | 0.08 | 0.17 |  |
|  | M2 | TA | C477T, A546A | 1.86 | 0.99 | 0.06 | 0.35 | | 0.10 | **0.0004** |  |
|  | M3 | CG | C477C, A459G | -0.49 | 1.13 | 0.67 | -0.02 | | 0.09 | 0.82 |  |
|  | M4 | TGT | C477T, A546G, T593T | -0.29 | 1.61 | 0.86 | 0.05 | | 0.08 | 0.51 |  |
|  | M5 | AC | C477A, C381C | -0.13 | 0.79 | 0.87 | 0.006 | | 0.05 | 0.91 |  |
| Streptococcaceae | Stre1 | G | C632G | -1.16 | 0.73 | 0.11 | 0.007 | | 0.09 | 0.94 |  |
|  | Stre2 | CT | C632C, C489T | 0.22 | 0.35 | 0.54 | 0.13 | | 0.05 | **0.003** |  |

**Table S2:** Oligotyping Output and Adjusted Analysis of the Association of Oligotype Abundances and the Vaccine Era Using Negative Binomial Regression (NBR) and Linear Mixed Effect (LME) Models. OT: Oligotype and SNVs: Single-Nucleotide Variants. The *E. coli* numbering for single nucleotide polymorphisms (SNPs) is based on an alignment with the *16S rRNA* sequence of *E. coli* O157:H7 str. Sakai strain. Indicated is the *E. coli* base, the position of the *E. coli* base, and the OT base according to the colors. We performed the NBR model with the relative abundance-based input matrix and the LME model with the binary-based input matrix in order to account for low-abundant OTs. Est: Estimate and Std.E: Standard Error. Baseline was the PCV7 era. PCV7 era: n=20 infants and n=355 samples, PCV13 era: n=21 infants and n=408 samples. Significant differences of the OTs between the two vaccine eras are indicated in bold.

*OT C2 is defined by T-, whereas the “-“ represents a gap in the original alignment. According to the *E. coli* sequence, this gap lies between position 472 and 473.

**Supplementary Table 3: Shannon Diversity Index of Clusters.**

| Cluster | SDI |
| --- | --- |
| Cluster1 | 0.31 |
| Cluster2 | 2.41 |
| Cluster3 | 0.95 |
| Cluster4 | 1.67 |
| Cluster5 | 1.08 |
| Cluster6 | 1.64 |
| Cluster7 | 1.33 |
| Cluster8 | 0.84 |
| Cluster9 | 0.88 |
| Cluster10 | 1.41 |

**Table S3:** Shannon Diversity Index (SDI) of Clusters. SDI was calculated using the *diversity* function of the *vegan* package in R based on the relative abundance of oligotypes within the clusters. Clusters contain each a different number of oligotypes – the higher the SDI, the more divers and the higher the evenness of the cluster. Figure 5 of the main manuscript gives detailed information on the composition of the clusters.
