# Supplementary material for: Influence of the pneumococcal conjugate vaccines on the temporal variation of pneumococcal carriage and the nasal microbiota in healthy infants: a longitudinal analysis of a case–control study
Source: Microbiome. 2017 Jul 24;5:85. doi: 10.1186/s40168-017-0302-6 (PMC5525364; doi:10.1186/s40168-017-0302-6)
Supplement: Supplementary file 2 — Suplementary Figure. (PDF 1873 kb) [file 40168_2017_302_MOESM2_ESM.pdf]

Additional file 2: Figure S2

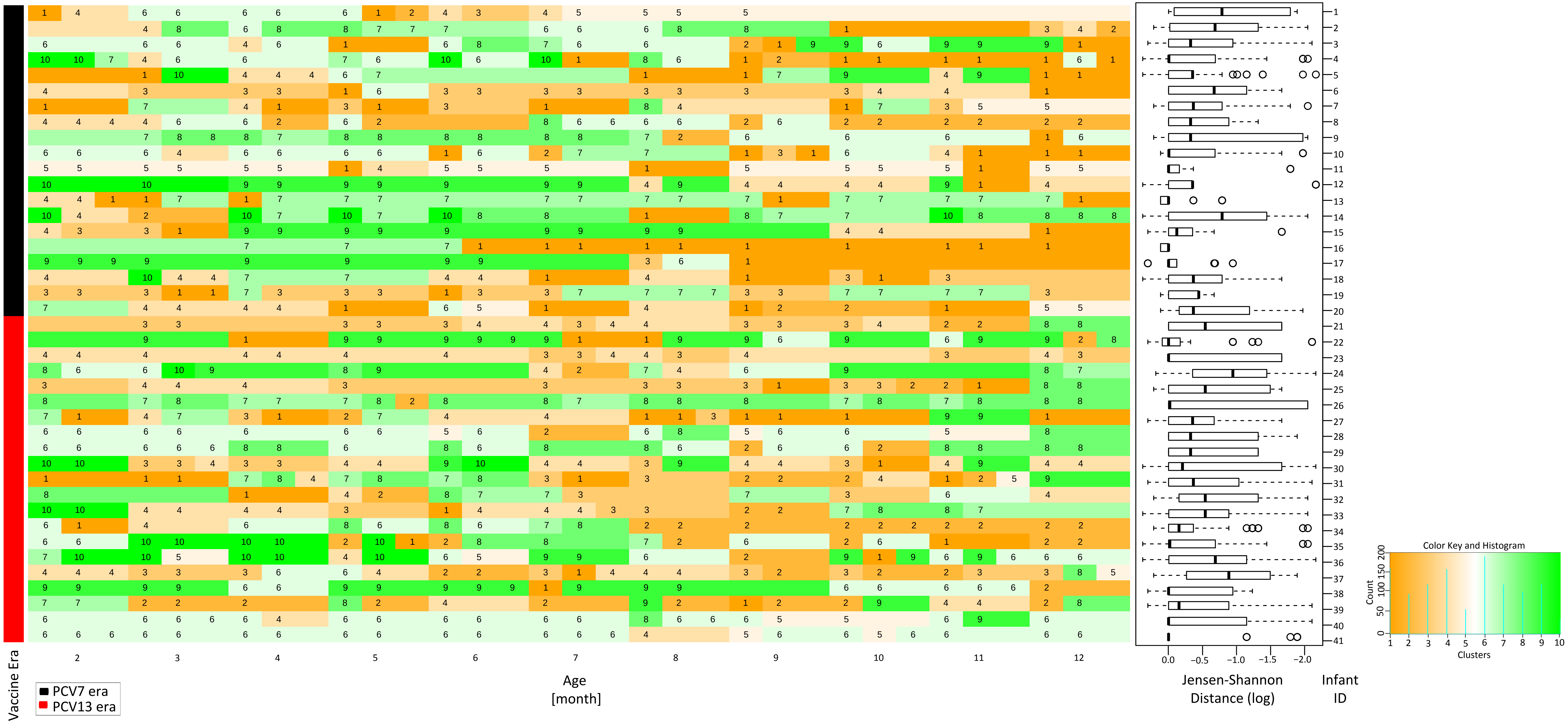

Additional file 2: Figure S 2: Dynamic of clusters in the 41 study infants within the first year of life. Figure indicates the cluster profile for each infant within the first year of life. Clusters are colored according to the color key on the bottom right. Each number indicates a sample and the corresponding cluster. The absence of a number indicates a missing sample. Vertical lines represent the time point of the PCV vaccination (first and second dose). Boxplots show the Jensen-Shannon distance (log transformed) between each pair of clusters within each infant.
